# Policy brief: ambient AI scribes and the coding arms race

**DOI:** 10.1038/s41746-025-02272-z

**Published:** 2025-12-24

**Authors:** Tinglong Dai, Joseph C. Kvedar, Daniel Polsky

**Affiliations:** 1 Carey Business School, Johns Hopkins University, Baltimore, MD USA; 2 Hopkins Business of Health Initiative, Washington, DC USA; 3 Department of Dermatology, Massachusetts General Hospital, Boston, MA USA; 4 Harvard Medical School, Boston, MA USA; 5 Bloomberg School of Public Health, Johns Hopkins University, Baltimore, MD USA

**Keywords:** Business and industry, Health care, Mathematics and computing, Medical research, Scientific community, Social sciences

## Abstract

Ambient AI “digital scribes” are rapidly moving into routine practice, easing documentation burden and physician burnout. Early evidence suggests these tools can increase billing and risk-adjustment coding intensity, prompting payer responses such as downcoding and risk-score recalibration. This Policy Brief contrasts their implications in fee-for-service and Medicare Advantage models, notes relevance for systems blending encounter-based and capitated payment, and outlines steps to preserve value without fueling a coding arms race.

## Ambient AI scribes: clinical promise and revenue implications

Ambient AI scribes—digital tools that listen to clinician–patient encounters and draft clinical notes—have moved from pilot projects into mainstream use at many large health systems. These tools promise to relieve physicians of tedious documentation and have shown early success in reducing burnout and after-hours “pajama time.” Independent evaluations confirm reductions in cognitive load and burnout^1,2^.

Yet adoption is no longer driven solely by well-being. The business case increasingly centers on revenue capture through more intensive coding. Ambience Healthcare’s July 2025 funding announcement, for instance, described its platform as “the leading ambient AI system for documentation, coding, and clinical documentation integrity,” highlighting how it “drives revenue-cycle performance”^3^. This language marks a clear pivot from earlier messaging about saving doctors time, signaling that ambient AI is now positioned as both a burnout remedy and a revenue engine—a shift that raises important questions about who ultimately benefits.

Competitive forces are accelerating this transition (Table 1). Doximity’s release of a free AI scribe signals that basic transcription is commoditizing^4^, shifting differentiation “after the transcript”—to how well products structure documentation that supports compliant, higher-complexity coding and comprehensive problem lists. Riverside Health in Virginia saw an 11% rise in physician work relative value units (wRVUs) and a 14% increase in documented Hierarchical Condition Category (HCC) diagnoses per encounter^5^. Northwestern Medicine clinicians using Nuance DAX billed more high-level Evaluation and Management (E/M) visits on average^6^, and a 2024 Texas Oncology study found that ambient scribes increased documented diagnoses from 3.0 to 4.1 per encounter^7^.

**Table 1:** Selected ambient AI scribe systems in current use

Vendor / product	Status (as of 2025)	Typical setting	EHR integration	Evidence cited in this brief
Abridge ambient AI for clinicians	Commercially available; deployed at multiple U.S. health systems	Outpatient primary care and specialties; expanding to inpatient settings	Integrated into Epic workflows via the Epic “Partners and Pals” / Abridge Inside collaboration; API-based integration with other enterprise EHRs	System-reported Riverside Health analysis noting an 11% increase in wRVUs and a 14% increase in documented HCC diagnoses per encounter^4^
Nuance Dragon Ambient eXperience (DAX) Copilot for Epic	Commercially available; deployed at multiple U.S. health systems and medical practices	Outpatient and specialty clinics; some expansion into other ambulatory settings	Native integration with Epic EHR; embedded ambient voice solution returning structured documentation directly into clinical workflows	Northwestern Medicine report and Microsoft/Nuance outcomes white paper describing reduced “pajama time,” improved documentation, and higher average level-of-service coding^5^
Doximity Scribe	Commercially available, HIPAA-compliant AI documentation tool; offered free of charge to verified U.S. physicians, NPs, PAs and medical students	Ambulatory visits across multiple specialties, used via the Doximity mobile and web apps	Compatible with multiple EHR systems; notes generated within Doximity platform and subsequently transferred into local EHR documentation workflows (no direct EHR integration)	Vendor announcement describing national rollout of a free AI scribe to Doximity’s clinician network^3^
Ambience Healthcare ambient AI platform	Commercially available, venture-backed ambient AI platform in use at several large systems	Outpatient, emergency, and inpatient settings across multiple specialties, focused on real-time documentation and coding support	Integrated with major EHRs; listed in Epic’s Toolbox as an ambient voice-recognition partner; supports coding-aware documentation at the point of care	Vendor- and system-reported outcomes highlighting reduced administrative burden and support for documentation, coding, and clinical documentation integrity (CDI)^2^

Collectively, these findings suggest that while ambient AI remains framed publicly as a tool for efficiency and burnout relief, its economic implications are increasingly difficult to ignore. It is against this backdrop that we compare how ambient scribes interact with U.S. fee-for-service and Medicare Advantage payment models (summarized in Table 2).

**Table 2:** Payment models explained

Payment model	How payment is determined	Scribe’s role	Payer countermeasures
Fee-for-service (FFS; including U.S. Original Medicare Parts A and B)	Clinicians are paid per visit or procedure based on Evaluation and Management (E/M) codes, and hospitals are paid per discharge based on diagnosis-related groups (DRGs).	Drafts thorough documentation so higher-level codes are justified	Claim audits and automatic downcoding of high-level visits
Medicare Advantage (MA)	Plans receive capitated payments adjusted for members’ risk scores (HCCs)	Captures all relevant diagnoses to increase risk scores	CMS risk-score adjustments and targeted audits
Key difference	FFS pays providers per service, so plan expenditures rise with billed visits, procedures, and hospitalizations; MA pays plans per enrollee using risk-adjusted capitation, so plan revenue rises with members’ risk scores even when provider payment remains FFS. Payers adjust formulas and audits to neutralize coding inflation.		

### Divergent incentives in fee-for-service and Medicare Advantage

This emerging revenue narrative raises two questions. First, can ambient AI improve the fidelity of documentation without distorting clinical priorities? Existing payment systems already influence clinical priorities and documentation, even without AI; the concern is whether ambient scribes amplify, mitigate, or reconfigure those distortions. Second, even if health systems see a short-term revenue bump, what happens once payers respond? Because incentives differ by payment model, we contrast Medicare Advantage (MA) and fee‑for‑service (FFS)—including U.S. Original Medicare Parts A and B—as illustrative examples (Table 2), noting that analogous distinctions between per‑encounter payment and risk‑adjusted capitation exist in other health systems as well.

On the first question, potential rises in wRVUs or HCCs do not necessarily mean upcoding; they often reflect previously omitted details now captured. From the provider’s viewpoint, capturing all legitimate billing complexity also helps offset the cost of ambient AI subscriptions. In the absence of direct reimbursement pathways, accurate coding becomes essential for sustaining adoption. Under-documentation is common: busy clinicians omit longstanding conditions, understate decision complexity, or skip the specificity coding rules require. Hospitals have long used electronic health record tools—such as Epic’s Best Practice Advisories (BPAs)—to remind clinicians to add diagnoses for risk adjustment^8^. At the policy level, the American Medical Association’s (AMA) Digital Medicine Payment Advisory Group is advising on coding and payment pathways for AI—including ambient AI scribes—and, given that practice expense is a major RVU component under AMA’s Resource-Based Relative Value Scale (RBRVS), how these costs are classified has become an important question for reimbursement design. From a payment perspective, ambient AI interacts with FFS and MA in different ways for providers and plans. In FFS (including Original Medicare and commercial fee-for-service), richer documentation tends to support higher-level E/M codes and additional billable services, so the revenue effect flows directly to clinicians and health systems. In MA, richer documentation primarily increases the plan’s risk-adjusted capitation payments by raising members’ risk scores; providers benefit only if their contracts with the plan share in that additional revenue (e.g., through capitation, shared savings, or risk- and quality-based bonuses).

What we mean by “upcoding” differs by market. In MA and other capitated, risk-adjusted arrangements, upcoding means documenting additional diagnoses (often HCCs) that raise risk scores and, in turn, payments to plans and—where contracts pass through some of that revenue—sometimes to providers. In FFS, it means billing a higher E/M level or more services based on documented complexity. Ambient AI can facilitate both: more complete diagnosis capture in MA and more support for higher-level E/M coding in FFS. Similar dynamics exist in DRG-based hospital payment, where more detailed documentation can shift discharges into higher-weighted DRGs. Related dynamics appear in other systems that adjust payments based on coded diagnoses or activity—for example, primary care commissioning in the English NHS—although the magnitude of payment differences and the scope for ambient scribes to shift revenue may be smaller.

### Payer responses and long-run equilibrium

The second question is where policy meets economics, especially in MA, where risk scores are tied to payments to plans. More complete documentation initially boosts risk‑adjusted capitation payments for MA plans, but regulators quickly adjust risk‑score formulas. As adoption widens, the financial advantage erodes and may even raise premiums for all. MA already applies coding intensity adjustments; if AI accelerates diagnostic capture, those offsets—and other countermeasures—will likely deepen. Evidence shows in-home risk assessments and chart reviews raise risk scores and payments—the patterns that prompted CMS to institute coding intensity adjustments^9^. Whether providers share in any temporary revenue gain depends on how they are paid by the plan—pure FFS contracts may see little direct impact, whereas capitated or shared-savings arrangements can transmit plan revenue gains to clinicians and health systems. If more complete documentation also prompts earlier or more appropriate treatment—for example, more proactive management of chronic conditions that are now reliably captured—ambient scribes could contribute to better outcomes and, in value‑based or prevention‑oriented systems, potentially lower long‑run costs rather than simply higher near‑term payments.

Payer responses will also play out in provider contracts. In FFS arrangements, health plans can tighten audits, deploy automated E/M downcoding tools, or cut base rates at renegotiation to offset documentation‑driven level increases, especially when outcomes do not improve. For example, starting in October 2025, Cigna began automatically reducing many level 4–5 E/M claims by one level unless documentation clearly supports higher complexity^10^, and Aetna Better Health has applied similar reviews^11^. Some providers may thus face blended effects: a near-term bump from richer documentation, followed by across-the-board offsets (in capitated programs) and contract-level rate recalibration (in FFS). In either case, late adopters may end up missing the temporary upside yet practicing under a lower baseline set after everyone else’s gains have been priced in.

### Who pays? Who gains?

Who ultimately pays for the potential rise in payments driven by ambient scribe technology? In the case of MA, taxpayers fund higher risk-adjusted payments to plans—and, where revenue is shared, to providers—until CMS adjustments catch up; in commercial FFS markets, employers and workers bear higher premiums until plans lower fees or downcode. Non-adopters may experience relative losses during the transition if baseline rates fall in response to industry-wide coding intensity. Vendors will have winners and losers; the winners will profit from subscription revenue and accumulated data assets. For clinicians and patients, the promise persists: less pajama time and a record that better reflects the encounter. The unresolved question is whether better coding translates into better care.

Distributional implications deserve attention. Large systems with integration teams, clinical documentation integrity (CDI) staff, and capital budgets can adopt and tune these tools fastest. Safety-net clinics and small practices may lag, either because subscription and workflow costs are real or because they are wary of compliance exposure. If baseline rates adjust downward while sophisticated adopters keep finding compliant documentation gains at the margin, the gap between resourced and under-resourced providers could widen. MA-heavy safety-net practices may be especially exposed: coding-intensity adjustments could claw back recent gains, leaving late adopters worse off. That is not an argument to halt adoption; it is a case for pairing diffusion with guardrails and targeted support.

### Governance and policy guardrails

What should those guardrails be? First, physicians and health systems must retain authorship: disable auto-accept and require active review of diagnoses and billing elements. Random audits comparing audio to signed notes can check drift toward “chart-stuffing.” Second, policymakers and large health systems could require transparency about AI-drafted content and certify tools that meet documentation quality standards. Such guardrails would protect against excessive note inflation while supporting appropriate use. Third, clinicians evaluating vendors should evaluate vendor claims against clinical and operational endpoints, not higher E/M levels or HCC capture. Fourth, health systems should exercise contract and pricing discipline: avoid overpaying in a race to match competitors; include clawbacks if payer offsets occur; protect data rights and avoid vendor lock-in; and benchmark against low-cost scribing to ensure one is paying for value beyond transcription. Fifth, clinicians in direct contact with patients should be transparent with patients. A clear, nontechnical disclosure that an AI assistant records to help the clinician document the visit—and that the clinician reviews and controls the note—can protect trust without derailing care^12^. Finally, payers and policymakers should align oversight with value. Audits should test medical necessity; in capitated programs, recalibrate risk models so payments track patients’ true need.

Ambient AI collapses the distance between care and coding more completely than any prior documentation tool. If revenue optimization becomes its defining purpose, we risk repeating a familiar cycle—an arms race that ends with higher administrative friction, payer pushback, and little improvement at the bedside. The equilibrium is still in flux. In the near term, some systems will capture real revenue gains; in time, commoditization and payer countermeasures will erode those advantages. Rather than accept a payer–provider standoff, regulators could make downcoding criteria transparent and appealable, while setting clear rules for AI-generated notes. Focused audits should target truly unjustified upcoding without penalizing completeness. These steps would align ambient AI with value-based care rather than a coming coding arms race.

## Data Availability

No datasets or custom code were generated or analyzed for this Policy Brief. All information is derived from previously published or publicly available sources cited in the reference list.
